# Cognitive and balance functions of astronauts after spaceflight are comparable to those of individuals with bilateral vestibulopathy

**DOI:** 10.3389/fneur.2023.1284029

**Published:** 2023-10-27

**Authors:** Gilles Clément, Olga Kuldavletova, Timothy R. Macaulay, Scott J. Wood, Deborah C. Navarro Morales, Michel Toupet, Charlotte Hautefort, Christian Van Nechel, Gaëlle Quarck, Pierre Denise

**Affiliations:** ^1^Université de Caen Normandie, INSERM, COMETE U1075, CYCERON, CHU de Caen, Normandie Université, Caen, France; ^2^KBR, Houston, TX, United States; ^3^NASA Johnson Space Center, Houston, TX, United States; ^4^Centre d'Explorations Fonctionnelles Oto-Neurologiques, Paris, France; ^5^Université de Paris Cité, INSERM U1141, Paris, France; ^6^Department of Otorhinolaryngology, Assistance Publique, Hôpitaux de Paris, Lariboisière Hospital, Paris, France

**Keywords:** bilateral vestibular loss, bilateral vestibular hypofunction, astronauts, vestibular tests, time perception, reaction time, spaceflight

## Abstract

**Introduction:**

This study compares the balance control and cognitive responses of subjects with bilateral vestibulopathy (BVP) to those of astronauts immediately after they return from long-duration spaceflight on board the International Space Station.

**Methods:**

Twenty-eight astronauts and thirty subjects with BVP performed five tests using the same procedures: sit-to-stand, walk-and-turn, tandem walk, duration judgment, and reaction time.

**Results:**

Compared to the astronauts' preflight responses, the BVP subjects' responses were impaired in all five tests. However, the BVP subjects' performance during the walk-and-turn and the tandem walk tests were comparable to the astronauts' performance on the day they returned from space. Moreover, the BVP subjects' time perception and reaction time were comparable to those of the astronauts during spaceflight. The BVP subjects performed the sit-to-stand test at a level that fell between the astronauts' performance on the day of landing and 1 day later.

**Discussion:**

These results indicate that the alterations in dynamic balance control, time perception, and reaction time that astronauts experience after spaceflight are likely driven by central vestibular adaptations. Vestibular and somatosensory training in orbit and vestibular rehabilitation after spaceflight could be effective countermeasures for mitigating these post-flight performance decrements.

## Introduction

After long-duration spaceflight, astronauts experience symptoms of vestibular dysfunction that are similar to those faced by patients with certain types of vestibular disorders, such as motion sickness (1, 2), dizziness or vertigo (3, 4), decreased ocular torsion (5, 6), delayed target acquisition during gaze shift (7), decreased stability during standing and walking (8, 9), inaccurate perceptions of self-orientation and motion (10, 11), and altered cognitive processing (12, 13). In addition, these challenges can cause increased weighting of or reliance on visual inputs (14, 15). After a few days, these functions return to baseline following a rapid exponential recovery curve (16, 17).

There is, however, a fundamental difference between the challenges to vestibular functioning caused by spaceflight and those of pathologically induced vestibular dysfunction (18). In normal conditions, the information from semicircular canals and otolith organs is congruent and is integrated to shape the representation of head movement and orientation in space. During exposure to the microgravity environment of spaceflight, astronauts receive normal information from their semicircular canals during head movement, but their otoliths provide information during head translation only (i.e., not during head tilt). After returning from space, the mechanism for integrating canal and otolith information must readapt to ground-based gravitoinertial force. Until this readaptation is complete, the astronauts experience spatial disorientation and postural incoordination (19). By contrast, both canal information and otolith information are disrupted in subjects with bilateral vestibulopathy (BVP) (20). This lack of information is substituted by sensory information from the visual and somatosensory systems and by compensation strategies (21).

This study compares the vestibular responses of astronauts during readaptation to Earth's gravitational force level after return from long-duration spaceflight to those of subjects with BVP, using the exact same test procedures. We hypothesized that the difference in the performance of the astronauts immediately after landing and the performance of BVP subjects would reveal the role of the compensation strategies used by the BVP subjects during rehabilitation.

## Materials and methods

### Astronaut subjects

Twenty-eight crewmembers (17 male members, 11 female members; 47.5 ± 6.7 years, mean ± SD) who flew for periods of 6–11 months (188 ± 58 days) on board the International Space Station participated in this study. Among them, 12 crewmembers were participating in their first spaceflight mission, whereas 16 crewmembers had completed one or several previous spaceflight missions of 6 months. All 28 crewmembers passed a United States Air Force Class III medical examination and had no known history of vestibular or oculomotor abnormalities.

The test procedures were approved by the European Space Agency Medical Board and the NASA Johnson Space Center Institutional Review Board and were performed in accordance with the ethical standards laid down in the 1964 Declaration of Helsinki. All subjects provided written informed consent before participating in the study.

### BVP subjects

Thirty BVP subjects (13 male subjects, 17 female subjects; 60.6 ± 13.0 years) were tested in the COMETE Laboratory at the University of Caen. The test procedures were approved by the French Ethical Committee (*Comité de Protection des Personnes de la Région Ouest I*, no: ID-RCB 2022-AO1513-40). Subjects were recruited by the *Association Française de Vestibulopathie Bilatérale* (www.afvbi.info).

Subjects had experienced BVP for 8 ± 2 years with no hearing loss or associated neurological symptoms. Before inclusion in the study, the subjects' BVP diagnoses were confirmed based on the absence of normal responses to a battery of neuro-otological tests performed in the Center d'Explorations Fonctionnelles Oto-Neurologiques (Paris) (20, 22). The function of the lateral semicircular canals was evaluated by measuring caloric nystagmus during a bithermal caloric irrigation of the right and left ears at both 44°C and 30°C. Twenty-nine subjects had a caloric nystagmus velocity of <6°/s (Table 1). The vestibulo-ocular reflex gain for each of the six canals was also evaluated during a video head impulse test with a video camera system. Most of the subjects (86.7–90%) had a deficit in this high-frequency test, with no compensatory eye movements and multiple catch-up saccades (Table 1). Utricular function was evaluated with ocular vestibular evoked myogenic potential (oVEMP), and saccular function was evaluated with cervical vestibular evoked myogenic potential (cVEMP). Twenty-three BVP subjects had altered utricular responses (oVEMP amplitude <100 microV). However, 16 subjects showed cVEMP responses, thus indicating that their saccular function was still operational. None of the BVP subjects had positional vertigo or showed signs of cerebellar ataxia, four subjects complained of oscillopsia, and five subjects reported having frequent migraines (Table 1).

**Table 1:** Number and percentage of subjects who tested positive for the neuro-otological tests used to diagnose bilateral vestibulopathy (BVP).

**Diagnostics tests**	**Criteria**	***N***	**%**
Right caloric nystagmus	< 6°/s	29	96.7
Left caloric nystagmus	< 6°/s	29	96.7
Right vHIT gain (three canals)	< 0.6	26	86.7
Left vHIT gain (three canals)	< 0.6	27	90.0
Right oVEMP	< 100 uV	23	76.7
Left oVEMP	< 100 uV	22	73.3
Right cVEMP	< 100 uV	14	46.7
Left cVEMP	< 100 uV	14	46.7
Positional vertigo	Present	0	0.0
Cerebellar ataxia	Present	0	0.0
Oscillopsia	Present	4	13.3.
Migraines	Present	5	16.7

vHIT, video head impulse test; oVEMP, ocular vestibular-evoked myogenic potential; cVEMP, cervical vestibular-evoked myogenic potential. According to the consensus document on the diagnostic criteria of the Classification Committee of the Barany Society (Strupp et al., 2017), BVP subjects must have bilaterally reduced angular VOR function documented by vHIT (gain < 0.6) and/or caloric (peak slow phase velocity < 6°/s) tests. All 30 subjects included in this study met these criteria.

### Experimental protocol

Five tests were administered: sit-to-stand, walk-and-turn, tandem walk, duration judgment, and reaction time. Due to time constraints, not all subjects of both groups performed all tests. Table 2 shows the number of subjects in each group who performed each test. The astronauts performed the tests ~3 months before launch (Astro Pre), monthly during the spaceflight (duration judgment and reaction time test only) (12, 19), and ~2 h after they returned to Earth (R + 0) and 24 h later (R + 1). Tests were performed on R + 0 and R + 1 because previous studies have shown significant changes in spatial orientation, eye movements, postural control, and gait during this period compared to preflight (23). In addition, post-flight testing timepoints are limited by operational constraints.

**Table 2:** Number of subjects that performed each test.

**Test**	**Astronauts**	**Timepoints**	**BVP subjects**
Sit-to-stand	25	Preflight, landing day	30
Walk-and-turn	13	Preflight, landing day	30
Tandem walk	25	Preflight, landing day	30
Duration judgment	10	Preflight, inflight, 1 day after landing	30
Reaction time	10	Preflight, inflight, 1 day after landing	30

During the sit-to-stand and walk-and-turn tests, the subjects wore a triaxial inertial measurement unit (IMU) (Opal V2 or Emerald, APDM Inc., Portland, OR, USA, for astronauts; X-Sens DOT; Xsens Technologies BV, Enschede, The Netherlands, for BVP subjects) attached to their trunk with an elastic band.

### Sit-to-stand

Subjects were requested to rise as quickly as possible from a seated position without using their hands and to maintain a quiet stance for 10 s. The time elapsed between the command to stand and the achievement of a stable posture was used as the measure of performance. The IMU data were used to determine when stable posture was achieved. The start and end of the stand were determined using the absolute angular velocity of the trunk pitch (19).

### Walk-and-turn

Following the sit-to-stand test above, subjects were asked to walk as quickly and safely as possible straight ahead toward a cone (4 m distance), walk around the cone, return, and sit in a chair. On the way to and from the cone, subjects stepped over a 30-cm high obstacle. This test was performed twice. The IMU data were used to calculate the time required to complete the obstacle course and the peak yaw angular velocity of the trunk while walking around the cone (19).

### Tandem walk

Subjects were instructed to walk 10 heel-to-toe steps with their arms folded across their chests and their eyes closed (two trials) or open (two trials). Each trial was recorded by video. Three reviewers independently examined the videos to determine the number of correct steps during each trial. A “misstep” was defined as any of the following: (a) the subject's stepping foot crossing over the plant foot; (b) the subject stepping to the side before completing the step; (c) the subject's stepping foot swinging in a wide, arcing path before stepping down; (d) a step duration >3 s; or (e) a gap larger than 10 cm between the heel of the front foot and toe of the back foot when the step was completed (8). The video order was randomized to minimize reviewer bias based on their awareness of the session. After all the reviewers had completed their assessments, the median value was used to determine the percentage of correct steps for each trial. A higher percentage of correct steps directly relates to better performance (19).

### Duration judgment

Subjects wore a head-mounted display (Oculus Rift, Oculus VR, Menlo Park, CA) and used a finger trackball connected to a laptop to report when 1 min had passed. Subjects wore earphones to listen to the instructions and to mask external noises. Using the finger mouse, they pressed a “go” button and waited for 1 min before pressing on a ‘stop' button. Only the “go” and “stop” buttons were displayed during the test. Subjects were not allowed to count the seconds passing by Navarro Morales et al. (12).

### Reaction time

Wearing the same head-mounted display used in the duration judgment test, subjects were required to press the finger mouse with their right hand as fast as possible in response to a stimulus (a blue square) that appeared for 50 ms in the center of the visual display at random intervals ranging from 1 to 3 s. The subjects performed 30 trials during each testing session, and the timings of the intervals were all different (24).

### Statistical analysis

The data were tested for normality with the Shapiro–Wilk test and for equality of variance with the Levene test. Most datasets did not meet the assumptions for parametric analysis, so a permutation test with 5000 repetitions was used. The statistical analysis was conducted in R Core Team (25) using the MKinfer package for permutation analysis (26). The p-value was adjusted for multiple comparisons with the false discovery rate (FDR) method. The significance threshold was set at a *p*-value of 0.05.

## Results

There were no statistical differences between the measures collected with the BVP subjects with and without saccular function, so the data of both groups were pooled together. The results obtained with astronauts have been reported in three previous studies (12, 19, 24). These results are summarized in this section and in the figures.

**Figure 1:**
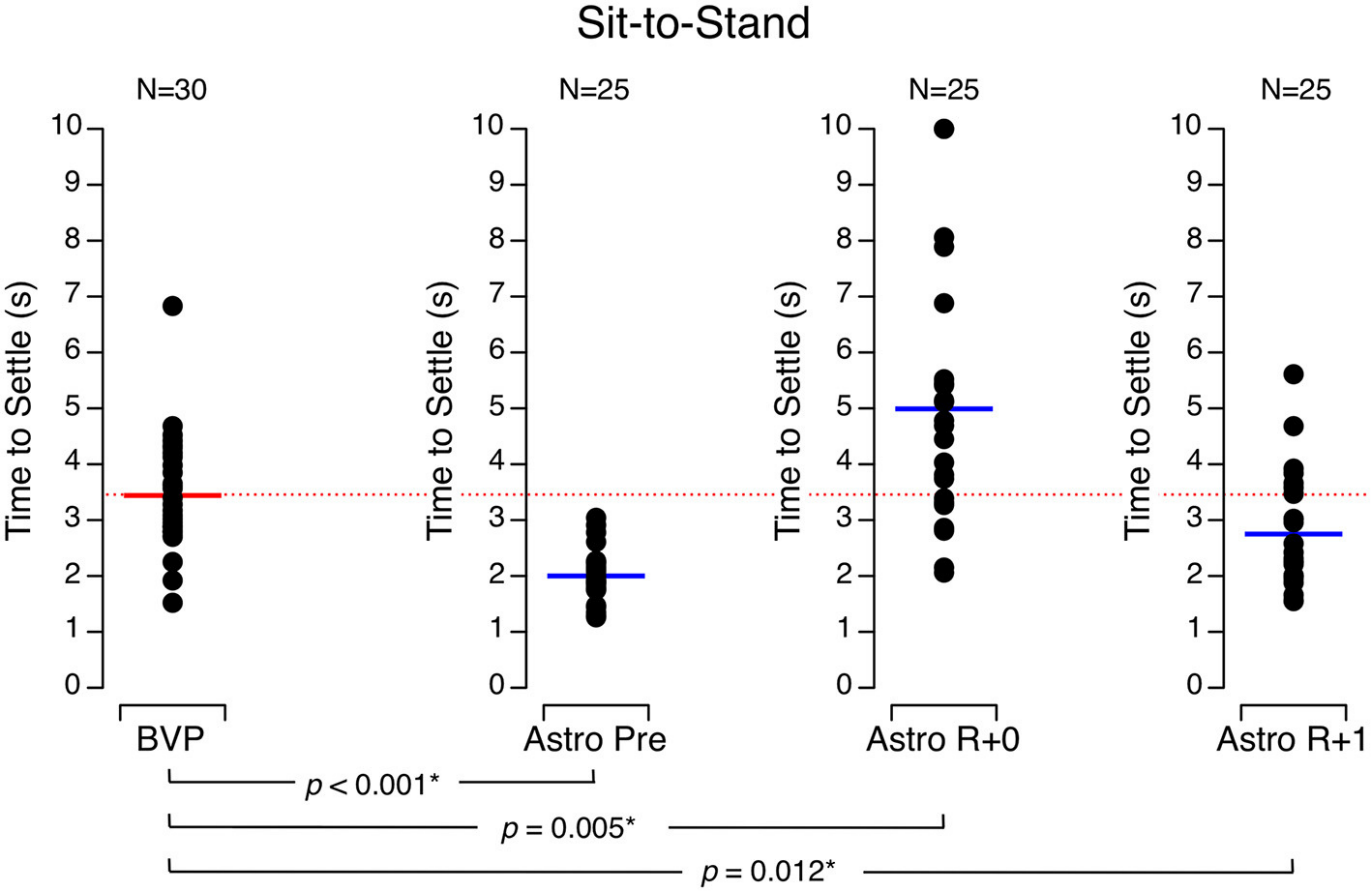
Time to settle during the sit-to-stand test. Black symbols are individual data. Red bar: mean for bilateral vestibulopathy (BVP) subjects; blue bar: mean for astronauts before spaceflight (Astro Pre), on landing day (Astro R + 0), and 1 day after landing (Astro R + 1). *p < 0.05.

The time required for astronauts to settle during the sit-to-stand test increased significantly on R + 0 (5.0 ± 2.2 s, mean ± SD) compared to values before flight (2.0 ± 0.5 s) and returned to baseline on R + 1 (2.7 ± 1.0 s). The time required for the BVP subjects to settle (3.4 ± 1.0 s) was significantly (a) longer than the astronauts' performance before flight, (b) shorter than the astronauts' performance on R + 0, and (c) longer than the astronauts' performance on R + 1 (Figure 1).

**Figure 2:**
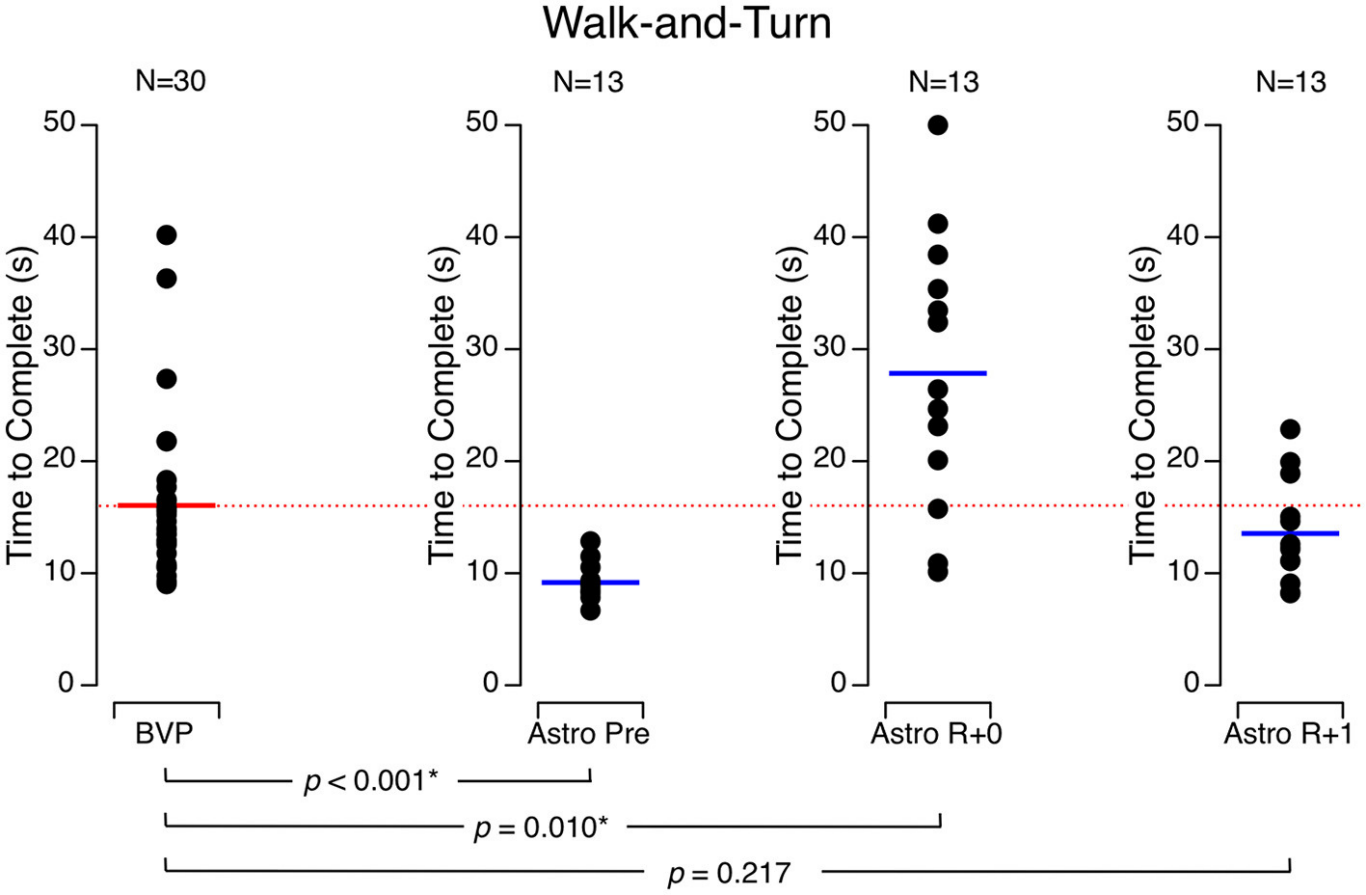
Time to complete the walk-and-turn test. Black symbols are individual data. Red bar: mean for bilateral vestibulopathy (BVP) subjects; blue bar: mean for astronauts before spaceflight (Astro Pre), on the landing day (Astro R + 0), and 1 day after landing (Astro R + 1). *p < 0.05.

**Figure 3:**
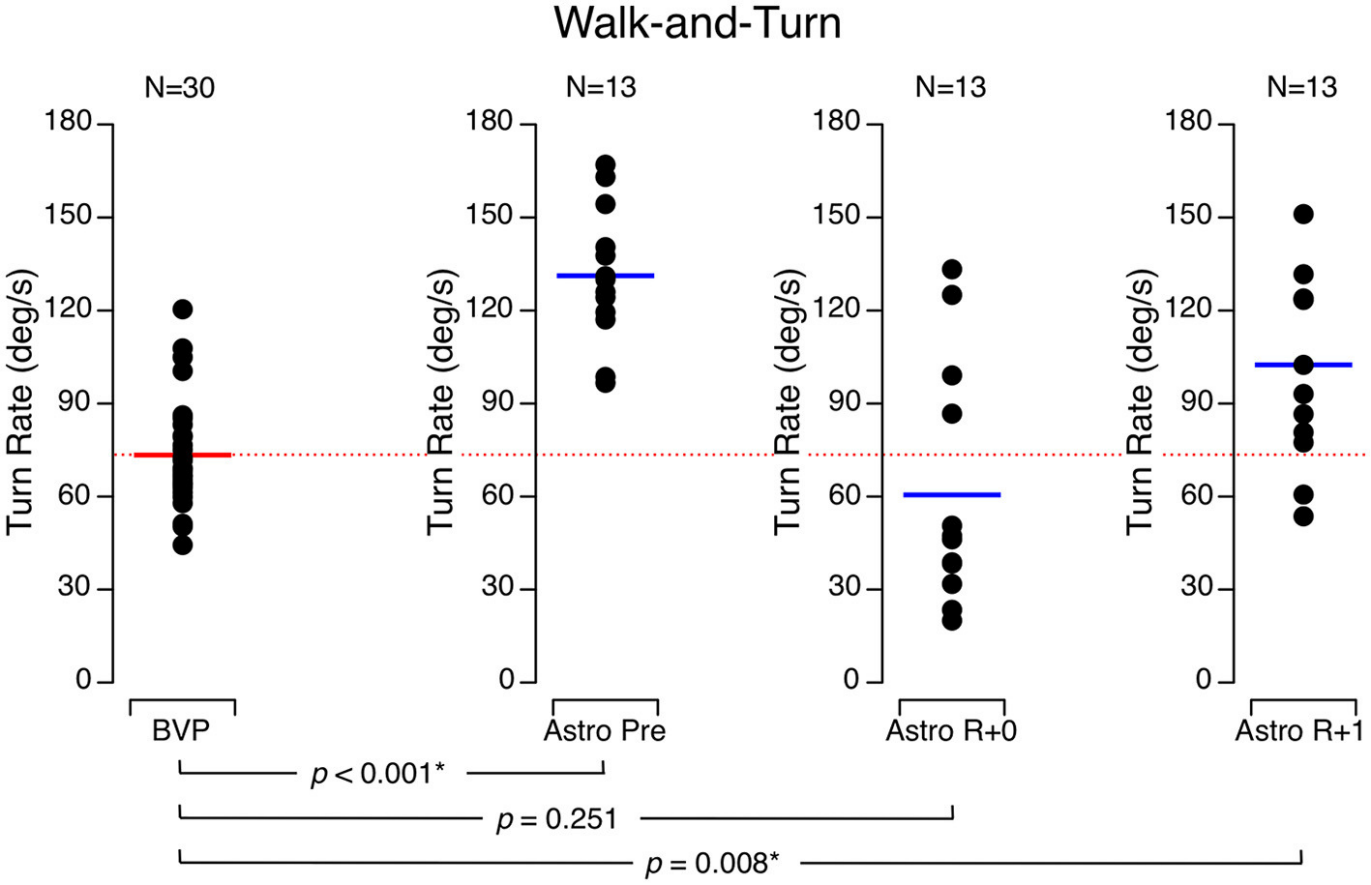
Turn rate around a cone (180 deg) during the walk-and-turn test. Black symbols are individual data. Red bar: mean for bilateral vestibulopathy (BVP) subjects; blue bar: mean for astronauts before spaceflight (Astro Pre), on the landing day (Astro R + 0), and 1 day after landing (Astro R + 1). *p < 0.05.

During walk-and-turn tests, the time required for the astronauts to complete the obstacle course was significantly longer on R + 0 (27.8 ± 12.0 s) than before flight (9.2 ± 1.6 s) and returned to baseline on R + 1 (13.6 ± 4.6 s). Similarly, the turn rate around the cone at the mid-course was significantly slower on R + 0 (60.5 ± 37.8 deg/s) and R + 1 (102.5 ± 29.7 deg/s) than it was before flight (131.2 ± 21.7 deg/s). The BVP subjects took longer to complete the obstacle course (16.1 ± 7.3 s) than the astronauts did before flight, but their speed was no different than the astronauts' speed on R + 1 (Figure 2). Moreover, the BVP subjects' trunk turn rate was slower (73.4 ± 17.6 deg/s) than the astronauts' rate before flight and on R + 1 but not significantly different from the astronauts' rate on R + 0 (Figure 3).

**Figure 4:**
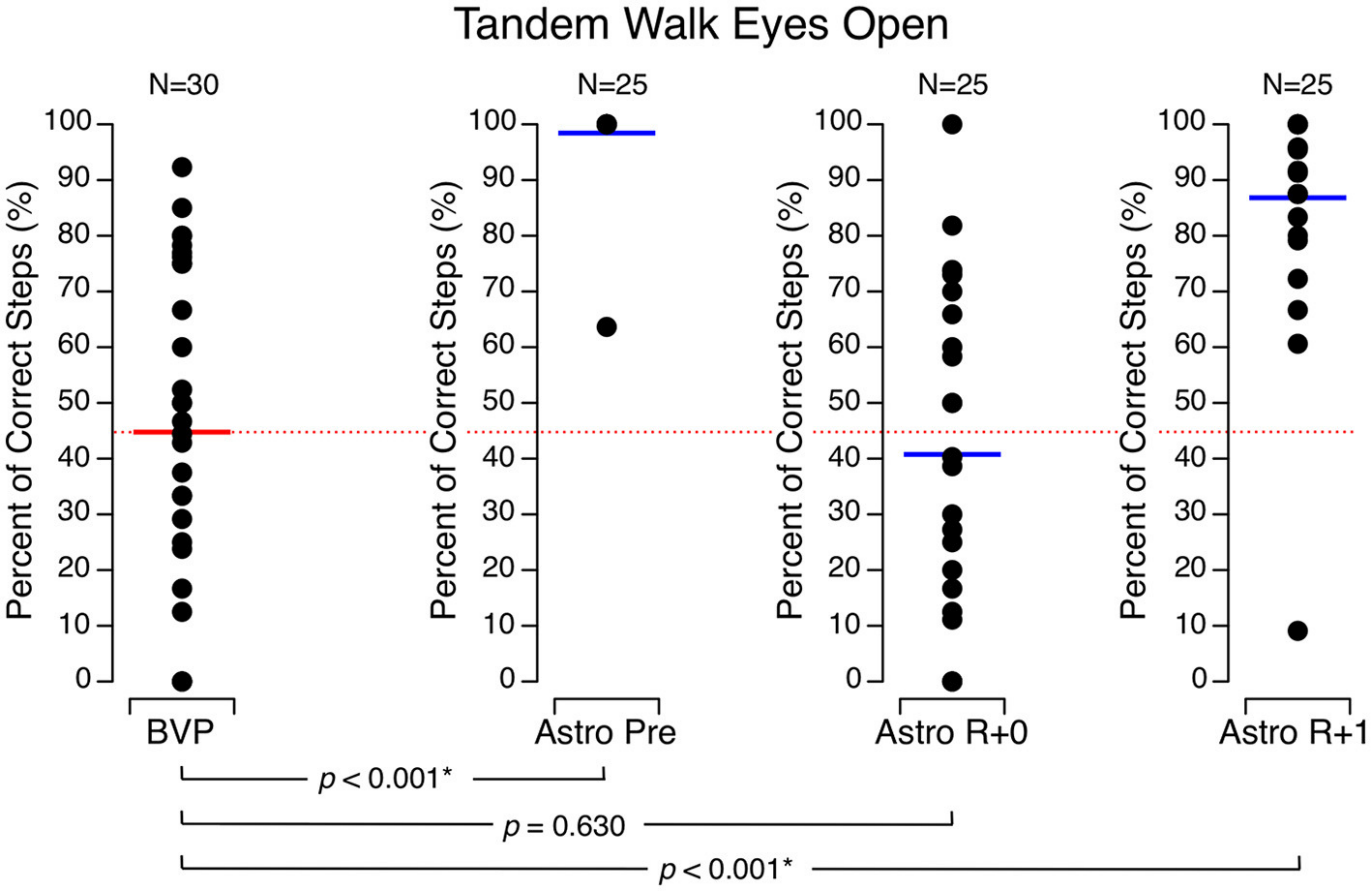
Percentage of correct steps during the tandem walk test with eyes open. Black symbols are individual data. Red bar: mean for bilateral vestibulopathy (BVP) subjects; blue bar: mean for astronauts before spaceflight (Astro Pre), on the landing day (Astro R + 0), and 1 day after landing (Astro R + 1). *p < 0.05.

**Figure 5:**
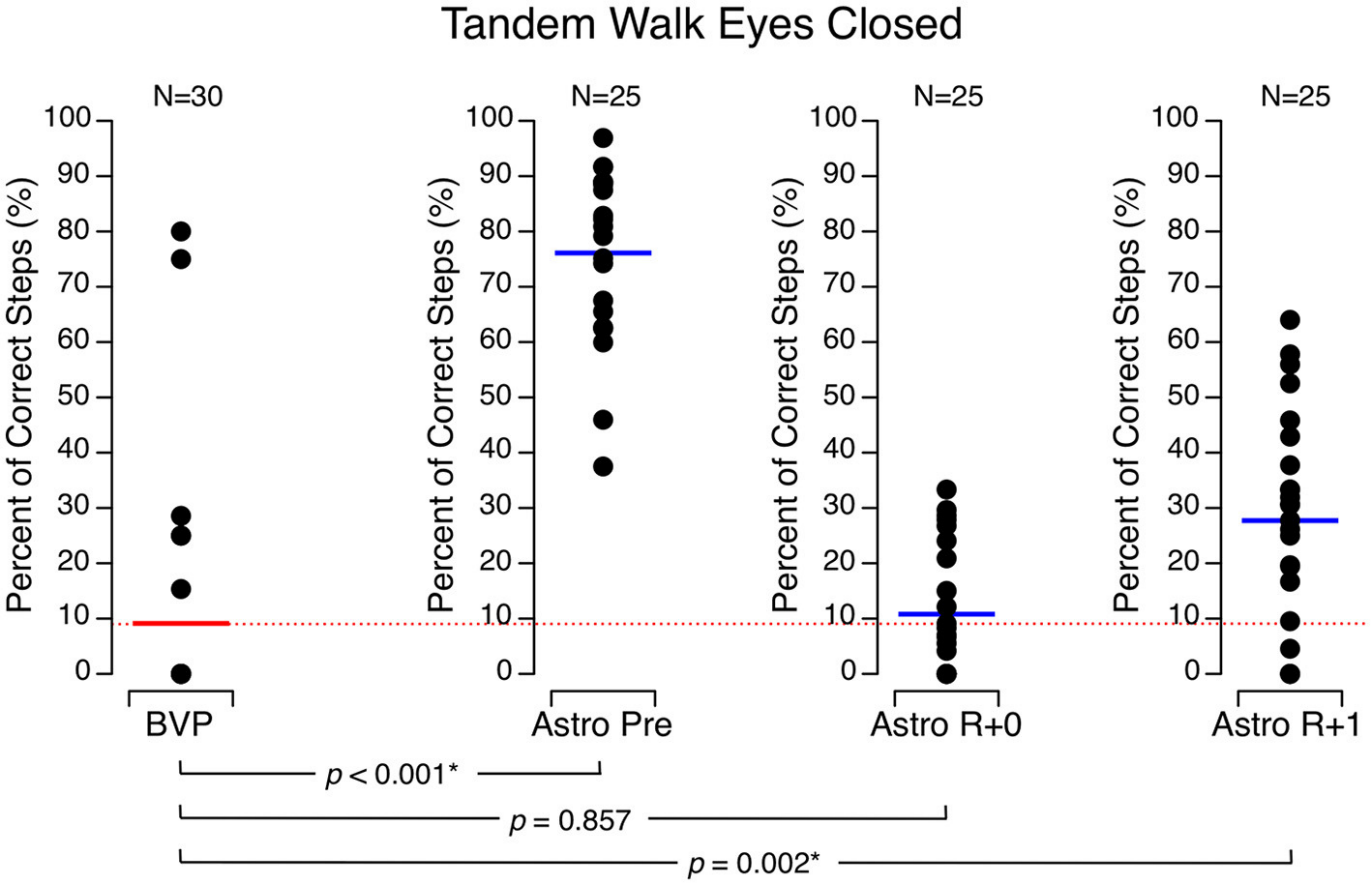
Percentage of correct steps during the tandem walk test with eyes closed. Black symbols are individual data. Red bar: mean for bilateral vestibulopathy (BVP) subjects; blue bar: mean for astronauts before spaceflight (Astro Pre), on the landing day (Astro R + 0), and 1 day after landing (Astro R + 1). *p < 0.05.

During the tandem walk tests with their eyes open, the percentage of correct steps in the astronauts was 98.4 ± 7.6 % before flight, 40.8 ± 30.0 % on R + 0, and 86.8 ± 20.1 % on R + 1. With their eyes closed, their percentage of correct steps was 76.1 ± 15.6 % before flight, 10.8 ± 11.8 % on R + 0, and 27.7 ± 19.3 % on R + 1. The BVP subjects achieved significantly less correct steps than the astronauts did before flight and on R + 1 both with their eyes open (44.8 ± 29.6 %) (Figure 4) and eyes closed (9.1 ± 20.7 %) (Figure 5). In both conditions, their performance was no different than the astronauts' performance on R + 0.

**Figure 6:**
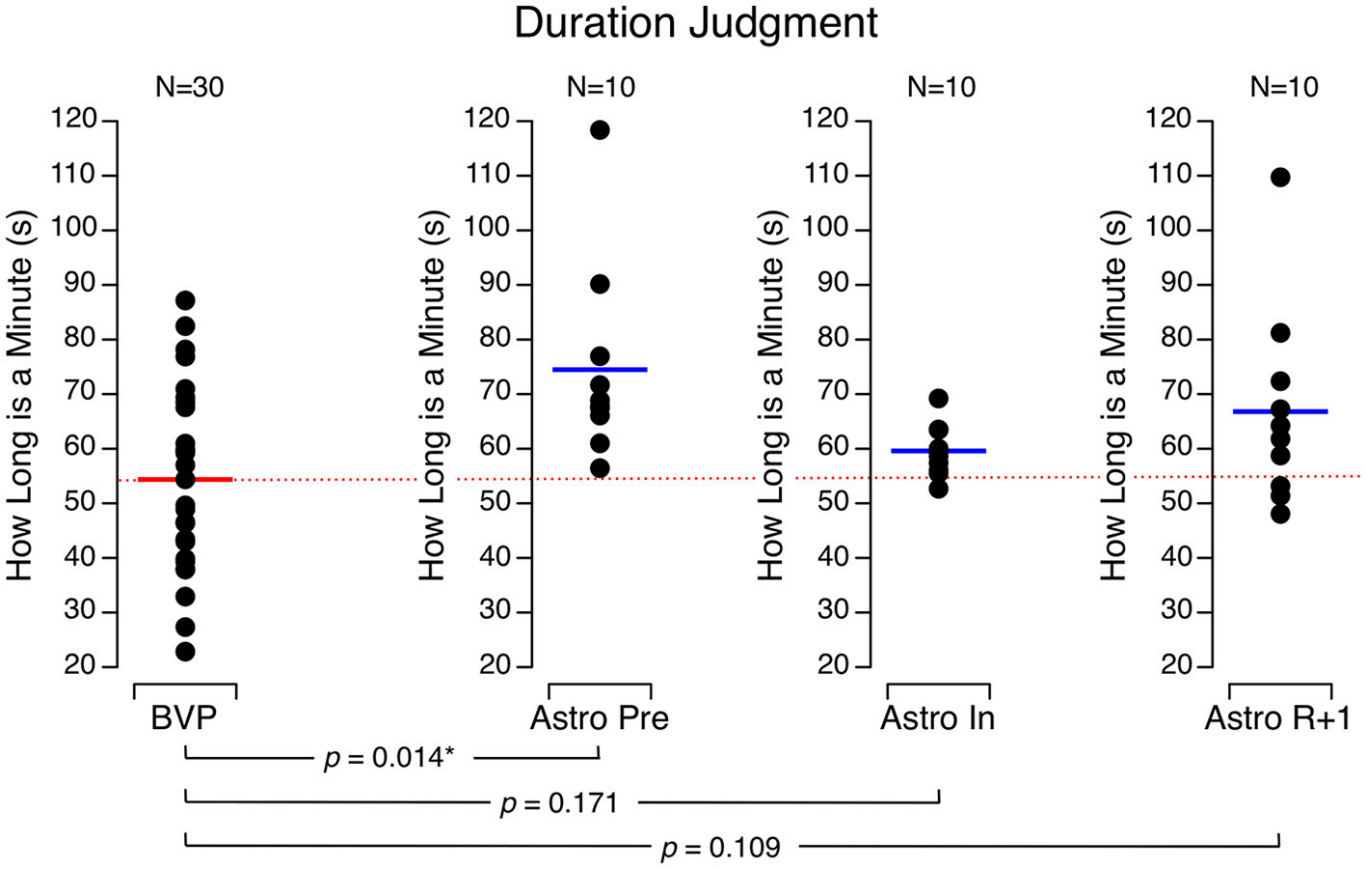
Perceived duration of 1 min. Black symbols are individual data. Red bar: mean for bilateral vestibulopathy (BVP) subjects; blue bar: mean for astronauts before spaceflight (Astro Pre), during spaceflight (Astro In), and 1 day after landing (Astro R + 1). *p < 0.05.

Before flight, the astronauts thought that 1 min had elapsed after 74.5 ± 17.9 s. The astronauts' perceived duration of 1 min decreased during flight (59.6 ± 4.8 s) and returned to baseline by R + 1 (66.8 ± 18.1 s). The BVP subjects perceived the duration of 1 min as 54.4 ± 17.0 s, which was significantly less than the astronauts' perceived duration before flight, but no different than the astronauts' performance during flight or on R + 1 (Figure 6).

**Figure 7:**
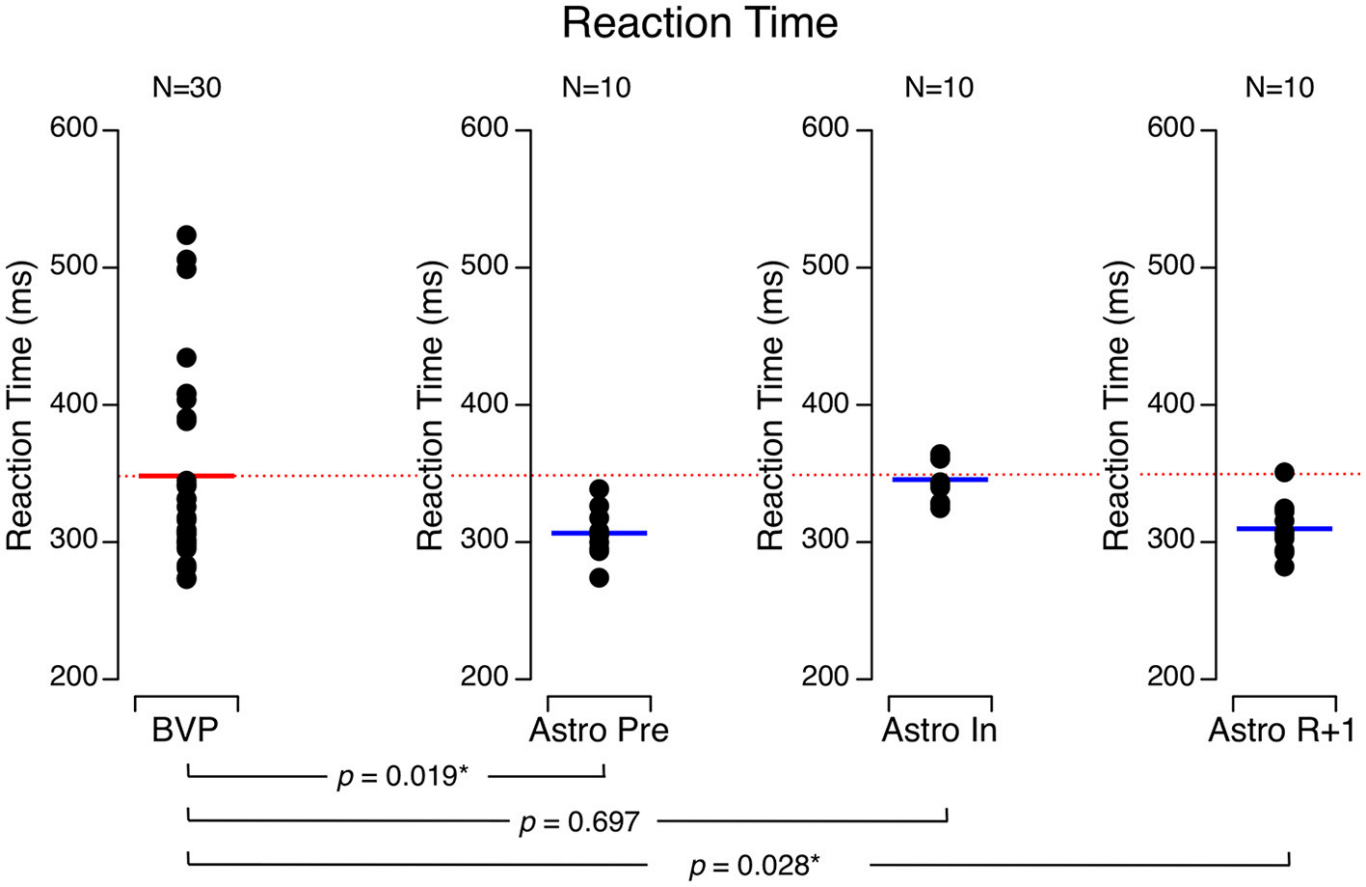
Reaction time to a visual target. Black symbols are individual data. Red bar: mean for bilateral vestibulopathy (BVP) subjects; blue bar: mean for astronauts before spaceflight (Astro Pre), during spaceflight (Astro In), and 1 day after landing (Astro R + 1). *p < 0.05.

The astronauts took more time to react to the presentation of a visual target during flight (346 ± 16 ms) than they did before flight (307 ± 18 ms), and their performance returned to baseline by R + 1 (310 ± 20 ms). The BVP subjects took significantly longer to react (348 ± 70 ms) to the target than the astronauts did before flight and on R + 1. However, the BVP subjects' reaction time was no different than the astronauts' performance during flight (Figure 7).

No significant differences were detected in responses of any of the tests for the astronauts participating in their first spaceflight mission and those astronauts who had flown a space mission previously.

## Discussion

Compared to the astronauts' responses before flight, the BVP subjects showed an impairment in all five tests. However, the BVP subjects' performance of the walk-and-turn and the tandem walk tests was comparable to that of the astronauts on R + 0. Moreover, the BVP subjects' time perception and reaction time were comparable to that of the astronauts during flight. The BVP subjects performed the sit-to-stand test at a level that fell between the astronauts' performance at R + 0 and R + 1.

One limitation of this study is that the astronauts reported in this study were not tested with the basic vestibular testing methods (i.e., caloric irrigation, vHIT, and vestibular evoked myogenic potential) after spaceflight as in the BVP subjects. Another limitation is that the BVP subjects did not experience nausea, but the astronauts showed motion sickness symptoms, especially on R + 0 (27). However, previous studies have shown that the vertical and roll VOR gain during head oscillations (28) and the ocular counter-rolling during head tilt (29) or centrifugation (5, 30) were decreased in astronauts immediately after landing. Although there is no report of VEMP after spaceflight in astronauts, cVEMP amplitude was found to be greater in microgravity during parabolic flight than in normal gravity (31).

Information from the vestibular (mainly otolith), proprioceptive, and somatosensory systems helps control movement of the body and the head when rising from a sitting position and when walking. We hypothesized that the impairment in astronauts' performance during these activities on R + 0 would be due to the malfunctioning of their otolith system due to adaptation to microgravity (5, 19). Indeed, it has been proposed that during adaptation to microgravity, otolith inputs are re-interpreted from head tilts to head translations. Therefore, when astronauts return to Earth, a head tilt relative to gravity would initially continue to be interpreted as head translation (32). This re-interpretation of otolith input impairs the performance of functional tasks that require accurate otolith cues. In agreement with this hypothesis, we observed that the performance of astronauts on R + 0 was comparable to that of the BVP subjects for most of the tests.

During the sit-to-stand test, however, the BVP subjects' performance was better than that of the astronauts on R + 0. Previous data have shown that postural equilibrium is altered after spaceflight or head-down tilt bed rest (8). Head-down tilt bed rest does not affect the information from the semicircular canals and otoliths. However, even though gravity is present during bed rest, the head-to-foot axial unloading reduces inputs to the somatosensory receptors of the feet along with those distributed throughout the body (33). The bed rest model can therefore be used to study how the somatosensory system contributes to changes in static balance. The BVP subjects in our study have presumably learned to substitute somatosensory information for the missing vestibular input, a mechanism that has been well demonstrated in both animal models and vestibular patients (34).

Furthermore, because the cardiovascular system is also challenged during the sit-to-stand test, the cardiovascular deconditioning observed after spaceflight and bed rest could also contribute to postural instability (8). It has been shown that BVP subjects can use proprioceptor-cardiovascular reflexes as a substitute for defective vestibulo-cardiovascular reflexes (35). Therefore, we infer that the larger decrement in the astronauts' postural stability on the landing day as compared to BVP subjects reflects the astronauts' combined changes in their vestibular, cardiovascular, and somatosensory systems. Inflight proprioceptive training has been proposed as a countermeasure for mitigating disorders in postural equilibrium and gait after spaceflight (36).

The tandem walk test was performed to assess changes in dynamic balance control. An impairment in the ability to maintain balance during tandem stance on rails has been observed in astronauts after short-duration spaceflights and after the Apollo missions to the Moon and in subjects after prolonged head-down bed rest (37, 38). Similarly, tandem heel-to-toe walking is significantly impaired after prolonged head-down bed rest and after spaceflight (8, 19). Our results showing similar performance decrements in astronauts on R + 0 and in BVP subjects (with both the eyes open and the eyes closed) suggest that altered vestibular inputs (the re-interpretation of otolith inputs due to spaceflight or the loss of vestibular inputs due to BVP) are the primary cause of the dysfunction in dynamic balance control. The limitations of this study are, however, the age difference between groups and the fact that the BVP subjects had received rehabilitation therapy (22).

We observed significant decrements in the time required to complete the obstacle course during the walk-and-turn test in the astronauts on landing day, which were comparable to those of BVP subjects. These decrements are presumably due to a shortening of step length, as has been reported by other studies of astronauts' performance of treadmill walking after spaceflight (39). Another factor that contributes to the longer time needed to complete the walk-and-turn test is the reduction in the angular velocity of the body during the turn around the cone. Previous studies have observed that velocity while walking around the corners of a triangular path was reduced in astronauts after returning from short-duration spaceflight (40).

In a previous study, we showed that healthy subjects, including astronauts before flight, estimated the duration of 1 min to be 74.1 ± 19.5 s (12). The estimates of the BVP subjects (55.4 s) were comparable to those of astronauts during their flight (59.6 s). The relative underproduction of a 1-min time period by the BVP subjects and astronauts refers to a relative overestimation of duration. In other words, these subjects feel that 1-min passes more quickly than clock time, i.e., time seems to go faster. We hypothesized that the change in time perception by the astronauts while in space could be due to reduced vestibular stimulations and slower motions (12), which is also the case in BVP subjects.

Altered time perception has been demonstrated in patients with schizophrenia (41), depression (42, 43), traumatic brain injury (44), attention-deficit/hyperactivity disorder (AHDH) (45), autism (46), Huntington's disease (47), multiple sclerosis (48), and Alzheimer's disease (49). However, this is the first time that an altered time perception has been characterized in vestibular patients. Evidence exists that vestibular stimulation alters time perception. For example, it has been shown that whole-body passive rotations affect the timing of sensory input (50) and the timing of motor responses as assessed with a paced finger-tapping task (51, 52). Moreover, growing, but still scarce, evidence exists of links between spatial processing and time perception (53).

A longer reaction time than healthy individuals has previously been observed in vestibular patients (54–56) and astronauts during spaceflight (24, 57–60), which confirms that the altered inputs from the otolith during exposure to microgravity could be responsible for the increase in reaction time seen in the astronauts.

Prior to the first human spaceflight, NASA and the U.S. Naval School of Aviation Medicine tested the response of 11 deaf men from Gallaudet University to various motion stressors (parabolic flight, human disorientation device, slow rotating room, and ships) to identify the role of the vestibular system in motion sickness. All these subjects had become deaf early in their lives due to spinal meningitis, which damaged the inner ear. The key findings from this research identified that subjects with non-functioning vestibular end organs showed no signs of motion sickness in rotating environments (61) or in severe storms at sea (62) and had a negligible change in performance associated with the slow rotation of a room (63). These observations are at the origin of the sensory mismatch hypothesis, the most widely accepted theory on motion sickness (64). Today, the space agencies are preparing human missions to the Moon and Mars. Once again, studies of vestibular patients help to elucidate the role of the vestibular system during the critical phases of these missions. Since the performance of the BVP subjects and astronauts is similar, BVP subjects could be used as test subjects in designing and testing the sort of tasks that astronauts might have to perform (or avoid) immediately upon being exposed to gravity.

The tests used in this study were selected to simulate functional tasks that a crewmember may be required to perform when they return to Earth or when they land on another planet, such as emergency capsule egress (65). These tests challenged balance control and cognition, i.e., functions that are vital for the performance of critical operational tasks immediately after landing on a planetary surface. The aerobic and resistance exercises performed on board the International Space Station do not fully protect against multisystem deconditioning (66). In addition, the results of NASA's field test study indicate that up to 15% of astronauts are not able to perform mission-critical tasks shortly after returning from spaceflight (17, 67). Therefore, a sensorimotor countermeasure that provides vestibular and somatosensory training may be required to ensure a safe level of balance control during the first hours after landing.

## Data availability statement

The raw data supporting the conclusions of this article will be made available by the authors, without undue reservation.

## Ethics statement

The studies involving humans were approved by the European Space Agency Medical Board, NASA Johnson Space Center Institutional Review Board, and Comité de Protection des Personnes de la Région Ouest (Tours, France). The studies were conducted in accordance with the local legislation and institutional requirements. The participants provided their written informed consent to participate in this study.

## Author contributions

GC: Conceptualization, Formal analysis, Investigation, Methodology, Writing—original draft. OK: Conceptualization, Formal analysis, Investigation, Methodology, Project administration, Writing—review & editing. TM: Conceptualization, Formal analysis, Investigation, Methodology, Writing—review & editing. SW: Conceptualization, Formal analysis, Funding acquisition, Investigation, Methodology, Project administration, Writing—review & editing. DN: Conceptualization, Formal analysis, Investigation, Methodology, Writing—review & editing. MT: Conceptualization, Formal analysis, Investigation, Methodology, Writing—review & editing. CH: Conceptualization, Formal analysis, Investigation, Methodology, Writing—review & editing. CN: Conceptualization, Formal analysis, Investigation, Methodology, Writing—review & editing. GQ: Conceptualization, Formal analysis, Investigation, Methodology, Writing—review & editing. PD: Conceptualization, Formal analysis, Funding acquisition, Investigation, Methodology, Project administration, Supervision, Writing—review & editing.
